# Molecular evidence for increased antitumor activity of gemcitabine in combination with a cyclin-dependent kinase inhibitor, P276-00 in pancreatic cancers

**DOI:** 10.1186/1479-5876-10-161

**Published:** 2012-08-08

**Authors:** Maggie J Rathos, Kavita Joshi, Harshal Khanwalkar, Sonal M Manohar, Kalpana S Joshi

**Affiliations:** 1 Oncology Franchise, Piramal Life Sciences Ltd, Mumbai, India; 2 Target Identification Group, Piramal Life Sciences Limited, 1-Nirlon Complex, Goregaon (E), Mumbai, 400 063, India

**Keywords:** Cdk inhibitor, P276-00, Gemcitabine, Combination studies, Pancreatic cancer

## Abstract

**Background:**

P276-00 is a novel cyclin-dependent kinase inhibitor currently in Phase II clinical trials. Gemcitabine is a standard of care for the treatment of pancreatic cancer. The present study investigated the effect of the combination of P276-00 and gemcitabine in five pancreatic cancer cell lines.

**Methods:**

Cytotoxic activity was evaluated by Propidium Iodide assay. Cell cycle and apoptosis was analyzed by flow cytometry. Genes and proteins known to inhibit apoptosis and contribute to chemoresistance were analysed using western blot analysis and RT-PCR. *In vivo* efficacy was studied in PANC-1 xenograft model.

**Results:**

The combination of gemcitabine followed by P276-00 was found to be highly to weakly synergistic in various pancreatic cancer cell lines as assessed by the combination index. Enhancement of apoptosis in PANC-1 cells and decrease in the antiapoptotic protein Bcl-2 and survivin was seen. P276-00 potentiated the gemcitabine-induced cytotoxicity by modulation of proteins involved in chemoresistance to gemcitabine and cell cycle viz. antiapoptotic proteins p8 and cox-2, proapoptotic protein BNIP3 and cell cycle related proteins Cdk4 and cyclin D1. The above results could explain the novel mechanisms of action of the combination therapy. We also show here that gemcitabine in combination with P276-00 is much more effective as an antitumor agent compared with either agent alone in the PANC-1 xenograft tumor model in SCID mice.

**Conclusions:**

The chemosensitzation of pancreatic tumors to gemcitabine would likely be an important and novel strategy for treatment of pancreatic cancer and enable the use of lower and safer concentrations, to pave the way for a more effective treatment in this devastating disease. Phase IIb clinical trials of P276-00 in combination with gemcitabine in pancreatic cancer patients are ongoing.

## Background

Pancreatic adenocarcinoma is one of the leading causes of cancer death with mortality rates almost identical to incidence rates [1]. Diagnosis usually occurs at late stages, making surgical intervention almost unfeasible due to low survival rates [2]. Standard treatments for advanced disease include radiotherapy and/or chemotherapy regimens. Radiotherapy has been shown to have some utility for regional confined cancers, but it is often too toxic for tissues surrounding the neoplasis. Widely used chemotherapeutic regimens include 5-fluorouracil (5-FU) and gemcitabine, a nucleoside analogue of cytidine (2’, 2’-difluorodeoxycytidine; dFdC) [3]. However, even gemcitabine, which is now considered the gold standard, has a response rate of less than 20%, although it does provide an improvement in the quality of life [4]. It is clear that novel therapeutic agents and/or combinations are needed for the treatment of pancreatic cancer.

Pancreatic ductal adenocarcinoma is characterized by mutations and/or silencing of tumor suppressor genes, such as *p53* and *Smad4*, the overexpression of mitogenic growth factors and their cognate high affinity tyrosine kinase receptors, and mutation of K-*ras*[5]. There are also defects in cell cycle-regulating genes, such as the increased expression of cyclin D1 and mutation/silencing of p16, which contribute to the inactivation of the retinoblastoma protein [6]. These alterations contribute to the excessive growth of pancreatic tumors and to the resistance of pancreatic cancers to chemotherapeutic agents [7].

Deregulated cell cycle progression has been considered as the hallmark of cancer progression, and therefore, is a practical target for anti-cancer drug development [8-11]. Cdks regulate the cell cycle progression, and their activity is increased in cancer cells. Accordingly, pursuits for the drugs that inhibit Cdks have been the intense area of research for last two decades, and numerous Cdk inhibitors have been identified. These drugs have been classified as pan-Cdk inhibitors or selective Cdk inhibitors [12]. The novel synthetic flavonoid P276-00 currently undergoing several Phase II clinical trials potently inhibits three specific cyclin-dependent kinase (Cdk) complexes, Cdk1/cyclin B, Cdk4/cyclin D1 and Cdk9/cyclin T. It exhibits an excellent anti-proliferative activity against panel of cancer cell lines including pancreatic adenocarcinoma cell lines and shows high efficacy in SCID mice tumour models [13,14].

Combination therapy is a major strategy for overcoming drug resistance and improving responses and cure rates. Cell cycle based agents have shown tremendous promise and potential against cancer; however, they are not fully effective by themselves. Similarly, cancer chemotherapies, which are the mainstream treatments for various human malignancies, are plagued by toxicity and the development of drug resistance, decreasing their overall clinical usefulness. Combining these two different categories of drugs has shown decreased toxicity and chemoresistance along with increased efficacy, suggesting that this could be an ideal approach to lower the cancer burden. Because P276-00 has potent antitumor activity in human pancreatic cancer cell lines and gemcitabine is currently used as first-line treatment for patients with locally advanced or metastatic cancer of the pancreas, the current work was undertaken to determine if they can be used in combination, thereby providing additive or synergistic benefits against pancreatic cancer. We thus evaluated the growth inhibitory potential of P276-00, gemcitabine and their combination in a panel of human pancreatic cancer cell lines and their *in vivo* antitumor efficacy against a human pancreatic cancer (PANC-1) xenograft.

## Methods

### Cell culture

Human pancreatic cancer cell lines, PANC-1, AsPC-1, MIA PaCa-2, BxPC-3 and Capan-1 were purchased from the American type Culture Collection, USA. The PANC-1 cell line was routinely cultured in MEM with NEAA (non-essential amino acids from SAFC biosciences), AsPC-1, MIA PaCa-2 and BxPC-3 in RPMI-1640 with 10% fetal bovine serum, Capan-1 in IMDM with L-glutamine and 20% serum and maintained as adherent cultures at 37°C in a humidified atmosphere containing 5% CO_2_.

### *In vitro* cytotoxicity assay

Cells were plated in 96-well cell culture plates (4 × 10^3^ cells/well) and were treated with P276-00 and /or gemcitabine at the indicated concentrations. Treated cells were maintained at 37°C in 5% CO_2_ for times indicated in the legends to the figures. A modified propidium iodide (PI) assay was used to assess the effects of the compounds on the growth of the human tumor cell lines [15]. Following continuous drug exposure, cell culture medium with or without drug was replaced by 200 μl of an aqueous PI solution (7 μg/mL). Because PI only passes leaky or lysed cell membranes, DNA of dead cells will be stained and measured, whereas living cells will not be stained. To measure the proportion of living cells, cells were permeabilized by freezing the plates, resulting in death of all cells. After thawing of the plates, fluorescence was measured using the POLARstar OPTIMA from BMG Technologies (excitation, 544 nm; emission, 620 nm), giving a direct relationship to the total cell number. IC_50_ values were determined by plotting compound concentration versus cell viability. The combination index (CI) was calculated by the Chou-Talalay equation, which takes into account both the potency and the shape of the dose-effect curve taking advantage of the Compusyn software (ComboSyn, Inc. NY, USA). The combination index is used for the quantification of synergism or antagonism for two drugs where CI < 1, =1, and >1 indicate synergism, additive effect, and antagonism, respectively.

### Cell cycle analysis

Cell cycle distribution was analyzed using propidium iodide (PI)-stained cells. Cells were cultured in 100-mm petri dishes and allowed to grow to 75-80% confluency. The cells are then treated with the drugs of interest at the indicated concentrations and time period and compared with control samples not exposed to drug. After drug exposure, cells were trypsinized, washed with PBS, resuspended and fixed with cold 70% ethanol. Samples were stored at −20°C before analysis. When samples are to be analyzed, they are centrifuged, the ethanol removed, and cell pellets washed twice with 1X PBS and resuspended in PBS containing 50 μg/mL PI and 50 μg/mL RNaseA. After incubation at room temperature for 20 min, cells were analyzed by flow cytometry using the Becton Dickinson (San Jose, CA) FACS Calibur flow cytometer.

### RNA extraction and reverse transcription-PCR

Total RNA extraction was performed from cultured cells using RNA easy kit (Qiagen, Valencia, CA) according to the manufacturer’s protocol, and purified RNA was quantitated and assessed for purity by UV spectrophotometry. cDNA was generated from 5 μg of RNA with Superscript III reverse transcriptase (Qiagen). The amplification of each specific RNA was performed in a 20 μl reaction mixture containing 2 μl of cDNA template, 1X PCR master mix, and the primers. The PCR primers used for detection of BNIP3 was from Superarray with annealing temperature of 60°C and cycle no. 22. Similarly for p8: Forward primer TAGAGACGGGACTGCG; Reverse primer GCGTGTCTATTTATTGTTGC with annealing temperature of 57°C and cycle no. 35. Cox-2: Forward primer TTCAAATGAGATTGTGGGAAAATTGCT; Reverse primer AGATCATCTCTGCTTGAGTATCTT with annealing temperature of 60°C and cycle no. 32. Tubulin Forward primer TCTGTTCGCTCAGGTCCTTTTGGCC; Reverse primer CGTACCACATCCAGGACAGA with annealing temperature of 55°C and cycle no. 22 were used. The PCR products were loaded onto 1.5% agarose gels and visualized with ethidium bromide under UV light. As a control for cDNA synthesis, reverse transcription-PCR was also performed using primers specific for β-actin gene.

### Immunoblot analysis

Protein extracts from PANC-1 cells, untreated or treated with P276-00, gemcitabine or both were prepared by suspending the cells in cell lytic buffer (Sigma) and Protease inhibitor cocktail (Sigma). The lysate was clarified by centrifugation to remove debris. For immunoblotting, each extract was prepared as above and an equivalent to 50 μg total protein was separated on SDS-PAGE, electrotransferred onto PVDF membranes. Membranes were probed with specific antibody to cyclin D1, Cdk4, Bcl-2, survivin (Santacruz Biotecnology), BNIP3 (Sigma), COX-2 (CalBiochem). Horseradish peroxidase conjugated anti-mouse or ant-rabbit IgG (Santacruz Biotechnology) was used to detect specific proteins. β-actin antibody (Sigma) was used as an internal control for protein loading. Detection of specific proteins was carried out with an enhanced chemiluminescence western blotting kit (Pierce) according to manufacturer’s instructions.

### *In vivo* studies

To examine the *in vivo* antitumor efficacy, PANC-1 cells (5 × 10^6^) cancer cells per mice were injected s.c. into 6 weeks old male SCID mice. Once the tumors attained a size of ~10 mm, animals were randomized to four experimental groups to receive vehicle control (saline) or P276-00 or gemcitabine or both. Gemcitabine was administered on day 1 and day 6 while P276-00 was administered once every day from day 2–5 and day 7–10. The first dose of gemcitabine was followed by P276-00 after an interval of 24 h, followed everyday with P276-00 for a total of five days, which comprised of one cycle. The next cycle would begin the next day. The treatment comprised of total 2 cycles. Body weight was recorded everyday. Tumor size and other signs of toxicity were recorded on every alternate day without sacrificing the mice. Tumor measurements i.e. the length and width of the tumors were measured using the vernier caliper. Tumor weight (mg) was estimated according to the formula for a prolate ellipsoid: {Length (mm) × [width (mm)^2^] × 0.5} assuming specific gravity to be one and π to be three. Tumor growth in compound treated animals is calculated as T/C (Treated/Control) × 100% and Growth inhibition Percent (GI%) was [100-T/C%].

## Results and discussion

### Effect of P276-00 and gemcitabine on cell proliferation

**Figure 1:**
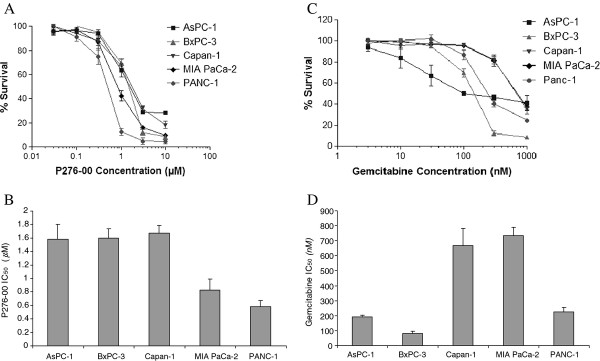
**The effect of P276-00 and gemcitabine treatment on growth of five pancreatic cancer cell lines.** Cells were seeded in 96-well plates and incubated overnight. P276-00 or gemcitabine was added at the indicated concentrations and cells were further incubated for 48 h. Cell proliferation was determined using PI assay. (**A** &**B**) The 50% inhibitory concentration of P276-00 in five different pancreatic cancer cell lines. (**C** &**D**) The 50% inhibitory concentration of gemcitabine in five different pancreatic cancer cell lines.

Cdk inhibitor P276-00 has been shown to induce cell cycle arrest and apoptosis of human tumor derived cell lines [13,14]. To elucidate the effects of the Cdk inhibitor P276-00 in pancreatic cancer cell lines, five pancreatic cancer cell lines AsPC-1, BxPC-3, Capan-1, MIA PaCa-2 and PANC-1 with varying genetic status and sensitivity to gemcitabine were treated with increasing concentrations (0–10 μM) of P276-00 for 48 h and cell proliferation was assessed by Propidium Iodide (PI assay). Treatment with P276-00 caused a dose-dependent decrease in the proliferation of all the five cell lines (Figure 1A). The results indicated that P276-00 was overall an effective inhibitor as a single agent for pancreatic cancer cell growth with both wild type and mutated K-ras. Except for BxPC-3 all the other 4 cell lines were K-ras mutated. MIA PaCa-2 and PANC-1 were more sensitive (2–3 times) than AsPC-1, BxPC-3 and Capan-1 as seen from the IC_50_ values (Figure 1B and Table 1). We also evaluated the effect of gemcitabine on cell growth *in vitro* and found that gemcitabine was effective in inhibiting cell growth of all the five cell lines (Figure 1C). BxPc-3 was highly sensitive to gemcitabine while AsPC-1 and PANC-1 was moderately sensitive. Capan-1 and MIA PaCa-2 were comparatively more resistant as seen from the IC_50_ evaluation (Figure 1D and Table 1).

**Table 1:** **IC **_**50 **_**values of P276-00 and gemcitabine in the different pancreatic cancer cell lines **

**Cell line**	**P276-00 IC**_**50**_**(μM)**	**Gemcitabine IC**_**50**_**(nM)**
AsPC-1	1.58 ± 0.22	190 ± 14.14
BxPC-3	1.6 ± 0.14	80 ± 14.14
Capan-1	1.67 ± 0.12	666.7 ±115.5
MIA PaCa-2	0.83 ± 0.16	733.3 ± 57.7
PANC-1	0.58 ± 0.09	225 ± 31.09

### P276-00 potentiates growth inhibition induced by gemcitabine in various human pancreatic cancer cell lines

**Figure 2:**
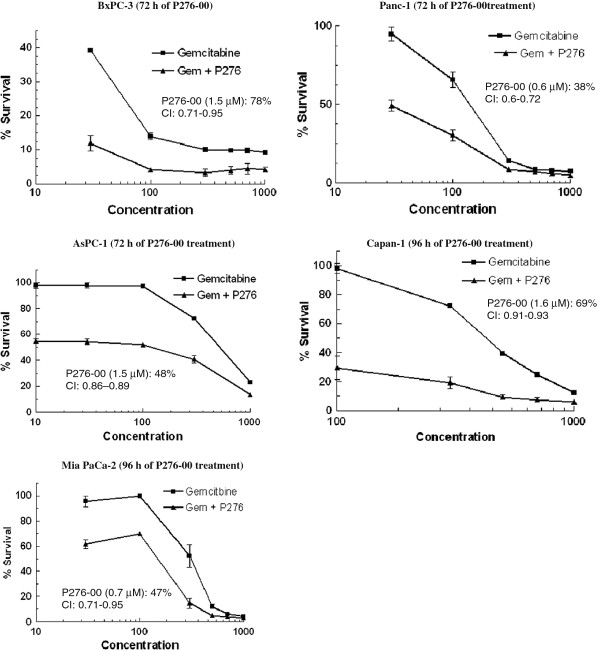
**Effect of P276-00 and gemcitabine used singly or in combination on survival of BxPC-3, AsPC-1, PANC-1, Capan-1 and MIA PACA-2 pancreatic cancer cell lines.** The cells were treated as described under Materials and Method section. There was significantly higher growth inhibition of the cells treated with gemcitabine (24 h) followed by P276-00 at IC_50_ concentration (1.5, 1.5, 1.6, 0.7 and 0.6 μM for AsPC-1, BxPC-3, Capan-1, MIA PaCa-2 and PANC-1 respectively) for 72 h or 96 h compared to the cells treated with either agent alone. P276-00 treatment alone in BxPC-3, AsPC-1, PANC-1, Capan-1 and MIA PaCa-2 showed 78%, 48%, 38%, 69% and 47% survival respectively.

The nucleoside analogue gemcitabine is commonly used in pancreatic cancer treatment regimens. However, gemcitabine is not curative in pancreatic cancer and treatment only increases survival by a few months [16]. To determine if P276-00 enhances the sensitivity of pancreatic cells to the growth inhibitory/apoptotic effect of gemcitabine, proliferation assays were done. For these studies, cells were either treated with P276-00 (corresponds to IC_50_ concentration at 48 h for each of the cell lines) or gemcitabine alone or in combination with serial concentrations of gemcitabine (10–1000 nM) followed by P276-00 for a period of 72 h or 96 h and viable cells were evaluated at 72 h or 96 h post treatment by PI assay. Combination treatment yielded significantly greater growth inhibition in a dose-dependent manner than either agent alone in all the cell lines tested (Figure 2). Treatment with P276-00 plus gemcitabine simultaneously was synergistic however, pretreatment of cells with P276-00 was antagonistic (data not shown). The combination index method developed by Chou and Talalay [17] were used to confirm and quantify the synergism observed with gemcitabine and P276-00. Combination index (CI) <1 is evidence for synergy, whereas CI >1 is evidence of antagonism, and CI = 1 indicates simple additivity of drug effect. The CI values of the combination of IC_50_ of P276-00 with various concentration of gemcitabine were calculated using CompuSyn software. BxPC-3 which is K-ras WT being the most sensitive cell line to gemcitabine was highly synergistic with P276-00 with CI values ranging from 0.38 –0.69 at all concentrations of gemcitabine ranging from 30 –1000 nM after 72 h of P276-00 treatment. The moderately sensitive cell lines to gemcitabine and K-ras mutated, AsPC-1 and PANC-1 were moderately synergistic with CI in the range of 0.86-0.89 and 0.6-0.72 respectively at concentrations of gemcitabine ranging from 30–300 nM after 72 h of P276-00 treatment. The two resistant cell lines to gemcitabine Capan-1 and MIA PaCa-2 were weakly to moderately synergistic with CI ranging from 0.9-0.93 and 0.71-0.95 respectively at concentrations of gemcitabine ranging from 500–700 nM after 96 h of P276-00 treatment. For all further studies, PANC-1 cell line which is moderately sensitive to gemcitabine and K-ras mutated was used as K-ras mutation is found in 90% of pancreatic cancers (Table 2).

**Table 2:** Synergistic range in different pancreatic cancer cell lines with varying genetic status

	**AsPC-1**	**BxPc-3**	**Capan-1**	**MIA PaCa-2**	**PANC-1**
K-ras	mut	wt	mut	mut	mut
p53	nk	mut	mut	mut	mut
p16	nk	-	-	-	-
P276-00	R	R	R	MS	MS
Gemcitabine	MS	S	R	R	MS
G + P276-00 (CI)	MSy	HSy	WSy	MSy	MSy
	(0.86-0.89)	(0.38-0.69)	(0.9-0.93)	(0.71-0.95)	(0.6-0.72)

***nk***: not known -: deletion ***mut***: mutated ***wt***: wild type.

***S***: sensitive ***MS***: moderately sensitive ***R***: resistant.

***WSy***: weakly synergistic ***MSy***: moderately synergistic ***HSy***: highly synergistic.

### P276-00 sensitizes PANC-1 cells to apoptosis induced by gemcitabine

**Figure 3:**
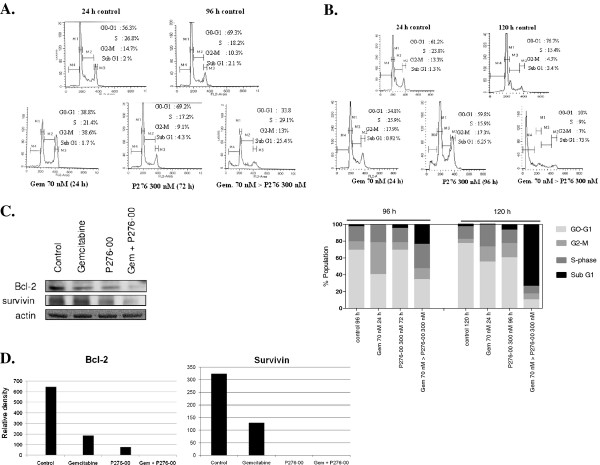
**The effect of P276-00, gemcitabine and the combination of gemcitabine followed by P276-00 on (A).** Cells were treated with 300 nM P276-00 for 72 h or 70 nM of gemcitabine for 24 h followed by only medium for additional 72 h or combination treatment of gemcitabine for 24 h followed by P276-00 for 72 h. (**B**) Cells were treated with 300 nM P276-00 for 96 h or 70 nM of gemcitabine for 24 h followed by only medium for additional 96 h or combination treatment of gemcitabine for 24 h followed by P276-00 for 96 h. Cell cycle analysis using Flow cytometry shows the percentage apoptosis in drug alone and combination treatment in comparison to untreated control. The percent distribution of cells in various phases of the cell cycle is represented as bar plot (**C**) Western blot analysis in PANC-1 cells of the antiapoptotic proteins Bcl-2 and survivin. Samples were obtained from PANC-1 cells treated with 300 nM of P276-00 for 72 h or gemcitabine for 24 h or the combination of gemcitabine for 24 h followed by P276-00 for 72 h. (**D**) Densitometric analysis of the Bcl-2 and survivin bands using the software Image J.

The above results were also confirmed by cell cycle analysis using flow cytometry wherein induction of apoptosis in PANC-1 cells treated with either P276-00 (300 nM) or gemcitabine (70 nM) alone or both was observed. Relative to single agents, gemcitabine (70 nM) pretreatment followed by P276-00 (300 nM) treatment for 72 or 96 h induced time dependent apoptosis in PANC-1 cell line as shown by cell cycle analysis using flow cytometry (Figure 3A and 3B). The percent distribution of the cells in various phases of the cell cycle is also depicted in the bar graph as shown in Figure 3B. The combination of P276-00 and gemcitabine showed highly significant apoptosis of 25.4% and 73% at 96 h and 120 h respectively as compared to either drug alone (*p* < 0.001). The expression of the antiapoptotic proteins Bcl-2 and survivin after treatment with P276-00 or gemcitabine or both was examined by Western blotting. As shown in the Figure 3C and the corresponding densitometry plot of the western bands in Figure 3D, the combination of P276-00 and gemcitabine decreased the expression of both antiapoptotic proteins Bcl-2 and survivin which may account for the drug synergy between P276-00 and gemcitabine.

### P276-00 regulates the cell cycle related proteins Cdk4 and cyclin D1 induced by gemcitabine

**Figure 4:**
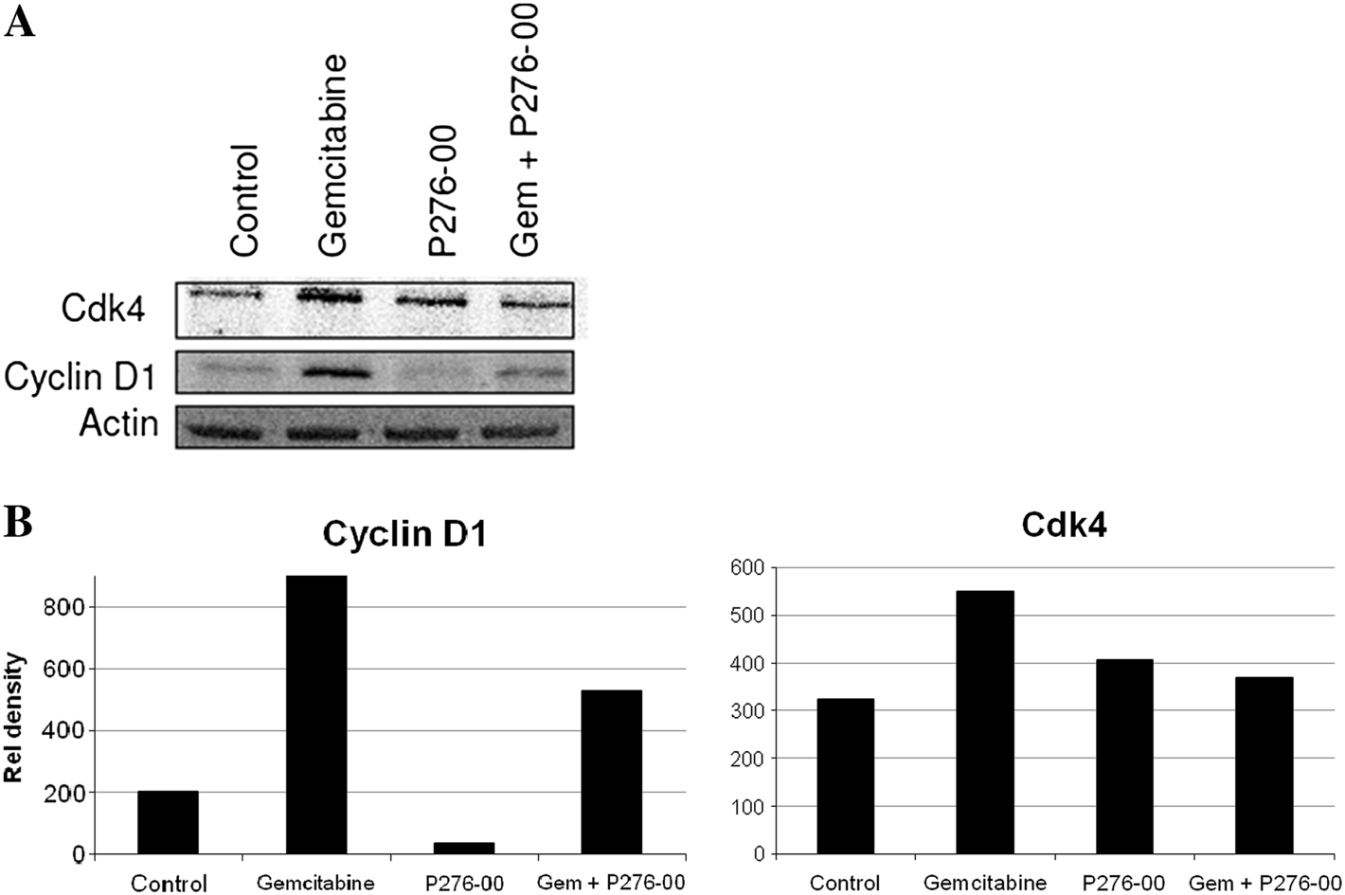
**Western blot analysis in PANC-1 cells of the cell cycle proteins Cdk4 and cyclin D1.** (**A**) Samples were obtained from PANC-1 cells treated with 300 nM of P276-00 for 72 h or 70 nM gemcitabine for 24 h or the combination of gemcitabine for 24 h followed by P276-00 for 72 h. (**B**) Densitometric analysis of the Cdk4 and cyclin D1 bands using the software Image J.

Next, we analyzed the effect of gemcitabine on Cdk4 and cyclin D1, which are necessary for cell cycle progression, and whether down regulation of these proteins by P276-00 a known Cdk inhibitor could be responsible for the more pronounced gemcitabine–induced apoptosis. Our results showed that relative to untreated control, gemcitabine treatment increased both Cdk4 and cyclin D1 levels as compared to control. Furthermore, the addition of P276-00 for 72 h to the cells post 24 h gemcitabine treatment reduced both cyclin D1 and Cdk4 protein levels which were upregulated by gemcitabine (Figure 4).

### P276-00 regulates genes responsible for chemoresistance to gemcitabine in PANC-1

**Figure 5:**
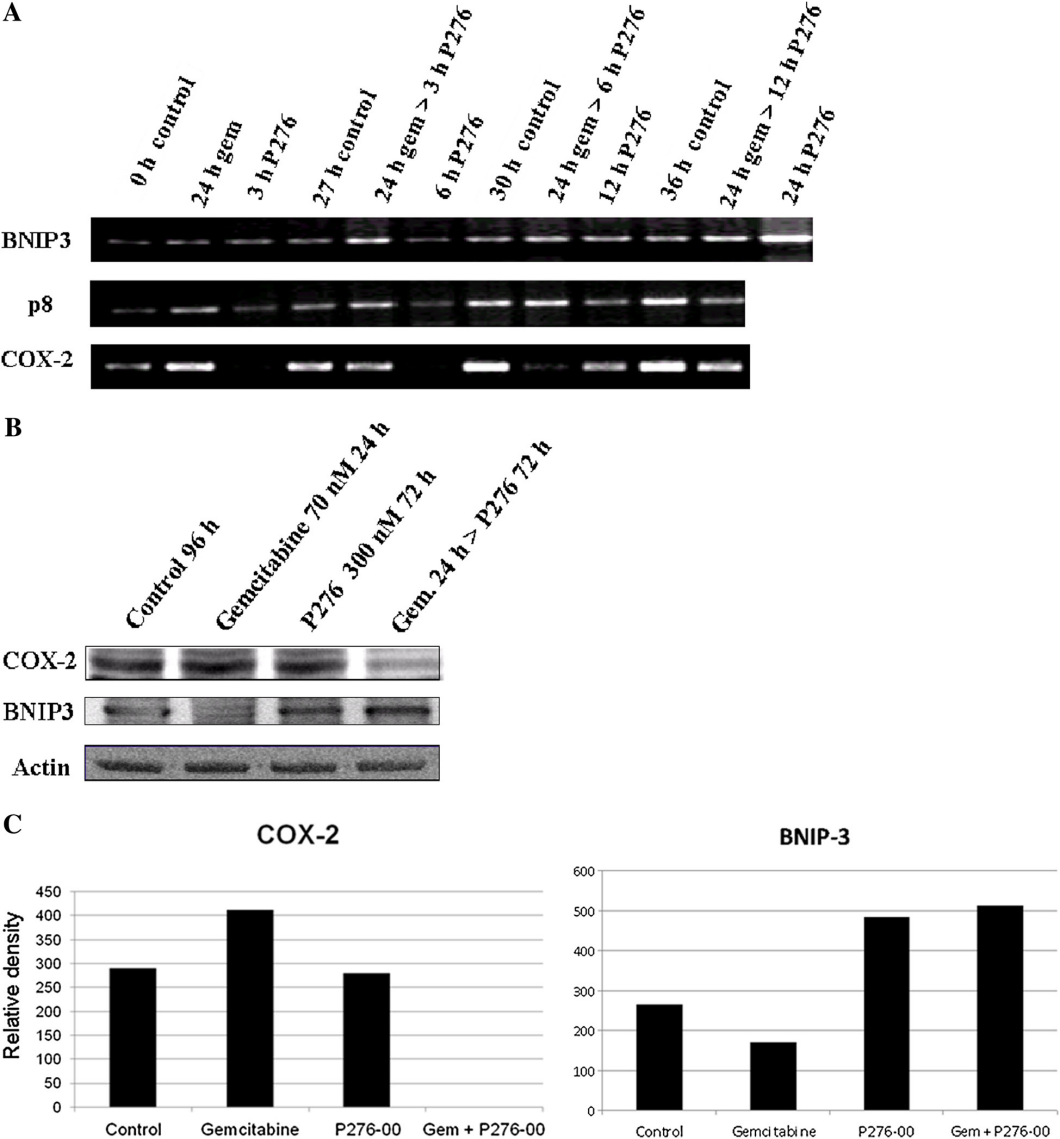
**RT-PCR and Western blot analysis in PANC-1 cells.** Samples were obtained from PANC-1 cells treated with 300 nM of P276-00 and/or 70 nM gemcitabine at various time points as indicated in the figure. (**A**) Electrophoretic analysis of BNIP3, p8 and COX-2 mRNA. (**B**) protein expression analysis of antiapoptotic protein COX-2 and proapoptotic protein BNIP-3. (**C**) Densitometric analysis of the COX-2 and BNIP3 bands using the software Image J.

Next we analyzed whether the genes responsible for chemoresistance to gemcitabine in PANC-1 could be regulated by P276-00. The three genes studied were the antiapoptotic genes COX-2, p8 and the proapoptotic gene BNIP3. The gene expression of all these genes was studied using RT-PCR in a time dependent manner (Figure 5A). The densitometry plots of the RT-PCR bands are shown in Additional file 1: Figure S1. Western blot analysis of BNIP3 and COX-2 proteins was studied at the end of the treatment for the combination and drug alone treatment (Figure 5B). The densitometry plots of the western bands are shown (Figure 5C). Our results indicated that relative to untreated control, the combination treatment of 70 nM gemcitabine followed by 12 h of 300 nM of P276-00 downregulated the gene expression of the antiapoptotic protein p8 and COX-2. The gene expression of the proapoptotic protein BNIP3 is upregulated in the combination by P276-00 from 3 h onwards. More interestingly it is significantly upregulated by P276-00 alone at 24 h. Western blot analysis also revealed that combination treatment inhibits antiapoptotic protein COX-2 and enhances the proapoptotic protein BNIP3.

### P276-00 potentiates anti-tumor effect of gemcitabine in xenograft model of pancreatic cancer

**Figure 6:**
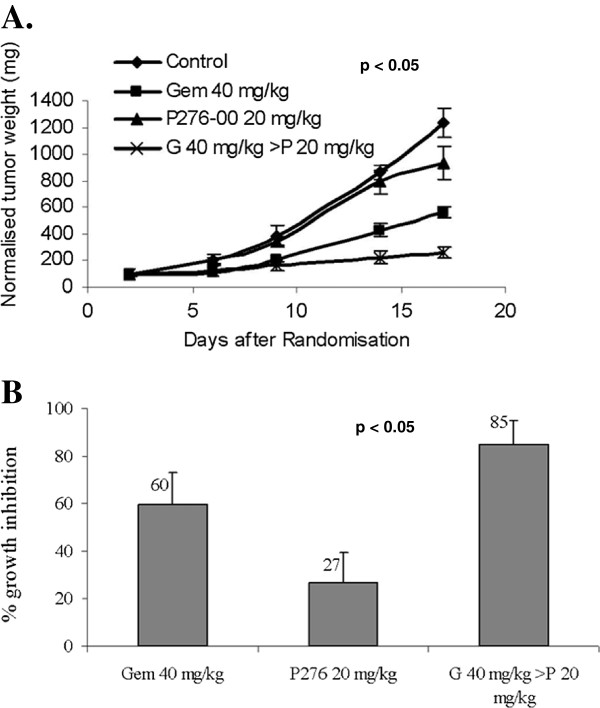
**The combination treatment of gemcitabine with P276-00 showed significant tumor growth inhibition in PANC-1 xenograft model.** (**A**) Differences in the Tumour weight (mg) in the different treatment groups. (**B**) Percent growth inhibition after treatment. Statistically significant difference (p < 0.005) of the combination treatment of gemcitabine and P276-00 compared with the control was seen.

The effects of the combination of P276-00 and gemcitabine on human pancreatic PANC-1 tumor xenograft were studied to determine if the synergy observed *in vitro* between P276-00 and gemcitabine also occurred *in vivo*. Treatment with either P276-00 (20 mg/kg) or gemcitabine (40 mg/kg) was initiated when tumors reached a size of ~10 mm in diameter. P276-00 and gemcitabine did not cause significant suppression of tumor growth, while the combination of the two drugs showed significant reduction in the mean tumor weight (Figure 6A). The growth inhibition in the combination treatment was 85% compared to control vs. 60% and 27% in gemcitabine and P276-00 treatment alone respectively (p < 0.05) (Figure 6B). No body weight loss was observed in both the combination and single drug treated groups indicating that the doses and schedule were well tolerated.

## Discussion

Despite rapid advances in diagnostic and operative techniques, pancreatic cancer remains one of the most difficult human malignancies to treat, which is partly due to the advanced stage of the disease and *de novo* chemoresistant behavior to cytotoxic chemotherapeutic agents and/or radiotherapy. In recent years, this problem has been addressed by combinatorial approach. Several randomized studies have shown significant increase in patient response rate by the use of combinations of different class of chemotherapeutic agents, but the major problem is due to treatment associated high toxicity, with no added benefit in significant overall survival [18-20]. However these limitations could be overcome by the use of rational chemotherapeutic combinations, in which toxic agents are used in lower doses, and the efficacy of treatment is complemented by using another safer agent that has a different mechanism of action. Hence in the present study we used a Cdk inhibitor P276-00 in combination with a commonly used chemotherapeutic agent, gemcitabine, to test its efficacy against a panel of pancreatic cancer cell lines differing in their genetic markers and sensitivity to the drugs used. The preclinical data indicates that P276-00 enhances the gemcitabine-induced cytotoxicity when added after 24 h of gemcitabine treatment as compared to either drug alone irrespective of the genetic status of the various pancreatic cell lines.

Increased apoptosis with the sequence of gemcitabine followed by P276-00 was shown by flow cytometry. Concentrations of gemcitabine and P276-00 used in this study resulted in minimal growth inhibitory effects when used alone. This concentration also resulted in negligible apoptosis as seen by the sub G1 phase in cell cycle analysis. However significant apoptosis of 25% and 73% was noticed when the gemcitabine treated cells were further exposed to P276-00 at 72 h and 96 h respectively at concentration that were suboptimal. The apoptosis in the combination was ~12 times more than P276-00 alone at 96 h of treatment. It is important to note that the above concentrations are easily achievable in humans; hence the *in vitro* results are expected to be relevant to future human studies. The increased apoptosis in the combination was also supported by the downregulation of the anti apoptotic proteins Bcl-2. Survivin, a member of the inhibitor of apoptosis (IAP) family is selectively expressed in the most common human neoplasms and appears to be involved in tumor cell resistance to some anticancer agents and ionizing radiation [21,22]. Specifically its antiapoptotic function seems to be related to the ability to directly or indirectly inhibit caspases and knockdown of the survivin gene expression seems to be a promising treatment strategy for cancer treatment. In this study we have shown that P276-00 and gemcitabine significantly decrease survivin levels as compared to either drug alone. Thus downregulation of Bcl-2 and survivin may be responsible for the increased apoptotic rate in the combination treatment of gemcitabine followed by P276-00 in PANC-1 cells.

P276-00 is a synthetic flavone and a cyclin-dependent kinase (Cdk) inhibitor in Phase I/II clinical trials at multiple centers. It has been shown to induce cell cycle arrest at G1 or G2/M, in association with direct inhibition of Cdk-1, -4, -6 and −9 [13]. Although the mechanisms of P276-00 induced antiproliferative activity have not been fully elucidated, inhibition of cyclin-dependent kinases may contribute to the anticancer effect, along with additional actions such as promotion of apoptosis, and a decrease of pRb and cyclin D1. Here we show for the first time that gemcitabine treatment of PANC-1 cells induces the cell cycle proteins cyclin D1 and Cdk4 and P276-00 when combined with gemcitabine, is capable of significantly reducing these increased protein levels of cyclin D1 and Cdk4 which are induced by gemcitabine treatment. Hence the reduction by P276-00 of these cell cycle proteins required for cell cycle progression may be one of the mechanisms by which the pancreatic cells are sensitized to apoptotic cell death by gemcitabine.

Resistance to the cytotoxic effect of gemcitabine can be related to multiple mechanisms including alteration of apoptosis regulating genes, alterations in the transport and cellular turnover of the drug as well as altered expression or sensitivity of enzyme targets. COX-2 appears to be of significance in pancreatic carcinogenesis. Several pancreatic cancer cell lines strongly express COX-2 and immunohistochemical studies show that 47-66% of human pancreatic tumours overexpress COX-2 relative to normal pancreatic tissue [23-26]. Besides COX-2 another antiaopototic protein p8 is strongly expressed in gemcitabine resistant cell lines and previous studies have shown higher expression of p8 in PANC-1 cells [27]. Not only are antiapoptotic proteins involved in gemcitabine resistance but proapoptotic proteins like BNIP3 are also involved in this chemoresistance. BNIP3, a member of the BH3-only subfamily of Bcl-2 family proteins, heterodimerizes and antagonizes the activity of prosurvival proteins such as Bcl-2 and Bcl-xl, thus promoting apoptosis. BNIP3 as a gene is strongly associated with intrinsic resistance to gemcitabine and frequently down regulated in pancreatic cancer. Also suppression of BNIP3 by siRNA reduced gemcitabine induced cytotoxicity in pancreatic cancer cells *in vitro*[28,29]. Gene and protein expression studies done by us, demonstrated that gemcitabine alone could induce p8 mRNA levels and both mRNA and protein levels for COX-2, resulting in reduced apoptosis supporting the notion that increased levels of these antiapoptotic proteins inhibits apoptosis. Similarly reduced levels of the proapoptotic protein BNIP3 also support this. In addition, our results showed that gemcitabine followed by P276-00 treatment downregulates gene and protein expression of the antiapoptotic protein COX-2, accompanied by downregulation of p8 gene expression and at the same time upregulates the gene and protein expression of the proapoptotic protein BNIP3.

Collectively, these results provide strong molecular evidence in support of our hypothesis that gemcitabine treatment followed by P276-00 could be useful in enhancing the gemcitabine-induced killing in pancreatic cancer cells by regulating the cell cycle proteins, antiapoptotic and proapoptotic proteins involved in gemcitabine resistance in pancreatic cancers. The data indicated that the synergy observed *in vitro* was extended to *in vivo* antitumour efficacy studies at well-tolerated doses and schedules. The results presented here have led to the initiation of Phase I/II clinical trials of combination of P276-00 and gemcitabine in pancreatic cancer patients.

## Conclusions

The present study describes the molecular mechanisms underlying the synergistic inhibition of pancreatic cancer cell growth *in vitro* by P276-00 and gemcitabine. Moreover, the significant *in vivo* antitumour activity by P276-00 and gemcitabine together with the absence of toxicity, provide a rationale basis for the development of novel therapies using the gold standard gemcitabine in combination with Cdk inhibitor in patients with advanced pancreatic cancer.

## Competing interests

The authors declare that they have no competing interests.

## Authors’ contributions

Conceptualization and designing of the above study was by KJ. MR and KJ did the combination experiments viz. cytotoxicity assays, gemcitabine resistant protein and gene expression profiling. SM has done cell cycle and apoptosis protein expression studies. HK has conducted *in vivo* experiments. Manuscript is written by MR and KJ. It is reviewed by all the authors.

## Authors’ information

Dr. Kalpana Joshi who has conceptualized this project has been working in the area of new drug discovery oncology for last twenty years. She is a lead inventor for two Cdk inhibitors viz. P276-00 and P1446A-05, and P2745-07, a Bcr-abl inhibitor which are currently undergoing clinical trials; and IGF-1R inhibitor where a preclinical candidate is selected for further development.

## Supplementary Material

Additional file 1 **Figure S1.** Densitometric analysis of the mRNA bands. Samples were obtained from PANC-1 cells treated with 300 nM of P276-00 and/or 70 nM gemcitabine at various time points as indicated in the figure. The figures show the relative densities of BNIP3, p8 and COX-2 mRNA bands shown in Figure 5A. Click here for file
