# Supplementary figures and images for: Molecular evidence for increased antitumor activity of gemcitabine in combination with a cyclin-dependent kinase inhibitor, P276-00 in pancreatic cancers
Source: J Transl Med. 2012 Aug 8;10:161. doi: 10.1186/1479-5876-10-161 (PMC3478973; doi:10.1186/1479-5876-10-161)

**Supplementary data**

**S1**

**
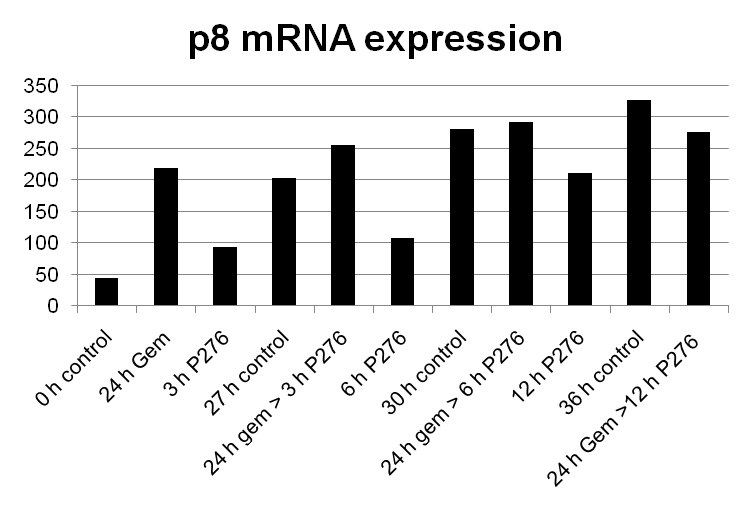
**


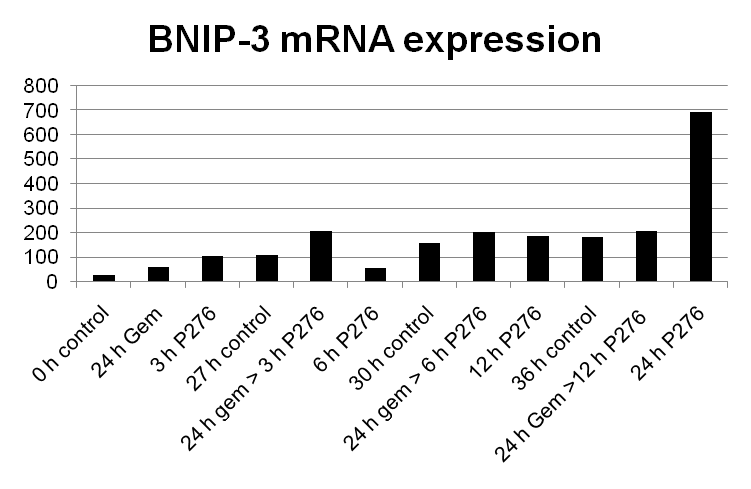


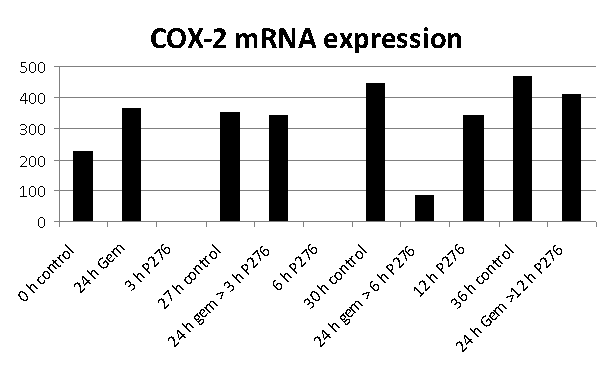

Supplement: Additional file 1 — Figure S1. Densitometric analysis of the mRNA bands. Samples were obtained from PANC-1 cells treated with 300 nM of P276-00 and/or 70 nM gemcitabine at various time points as indicated in the figure. The figures show the relative densities of BNIP3, p8 and COX-2 mRNA bands shown in Figure 5A. [file 1479-5876-10-161-S1.doc]
